# A novel adaption to suction-assisted seroma aspiration

**DOI:** 10.1308/rcsann.2024.0068

**Published:** 2024-11-21

**Authors:** MA Langford, W Chow, P Kalu, J Birch

**Affiliations:** Oxford University Hospitals NHS Foundation Trust, UK

## Background

Seroma formation is a common sequela of lymph node dissection and autologous breast reconstruction.^1^ Simple syringe aspiration of a seroma can be cumbersome and physically tiring, and has led to the description of augmented techniques.^2,3^ Here, we document our modification of a previously described technique to aid in the location, drainage and, if required, sampling of seroma fluid.^3^

## Technique

**Figure 1:**
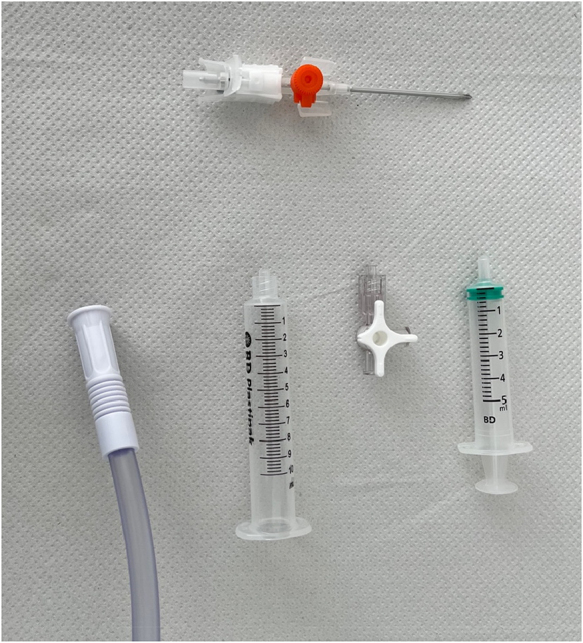
Constituent pieces required for the technique. Clockwise from top: 14G cannula, 5ml syringe, three-way tap, 10ml syringe with plunger removed and suction tubing.

The seroma is assessed clinically and an ideal site for puncture is identified, with or without an ultrasound scan. The equipment used for this is shown in Figure 1. Using an aseptic technique, a 14G cannula is placed on one end of a three-way-tap. The second port is connected to a 5ml syringe, and the third port is connected to a 10ml syringe, with the plunger removed to allow the end of simple suction tubing to be placed snugly in the lumen.

**Figure 2:**
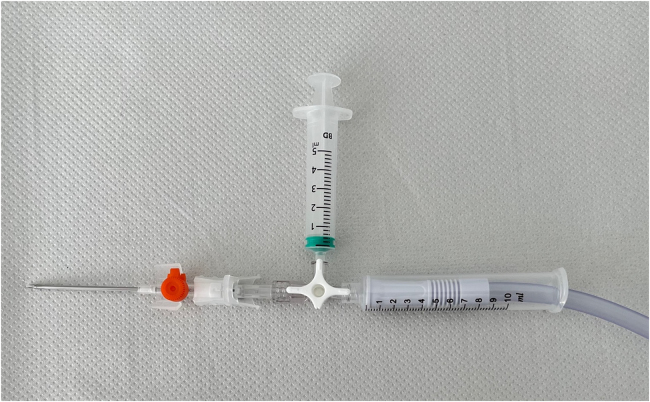
Constituent parts assembled for use

**Figure 3:**
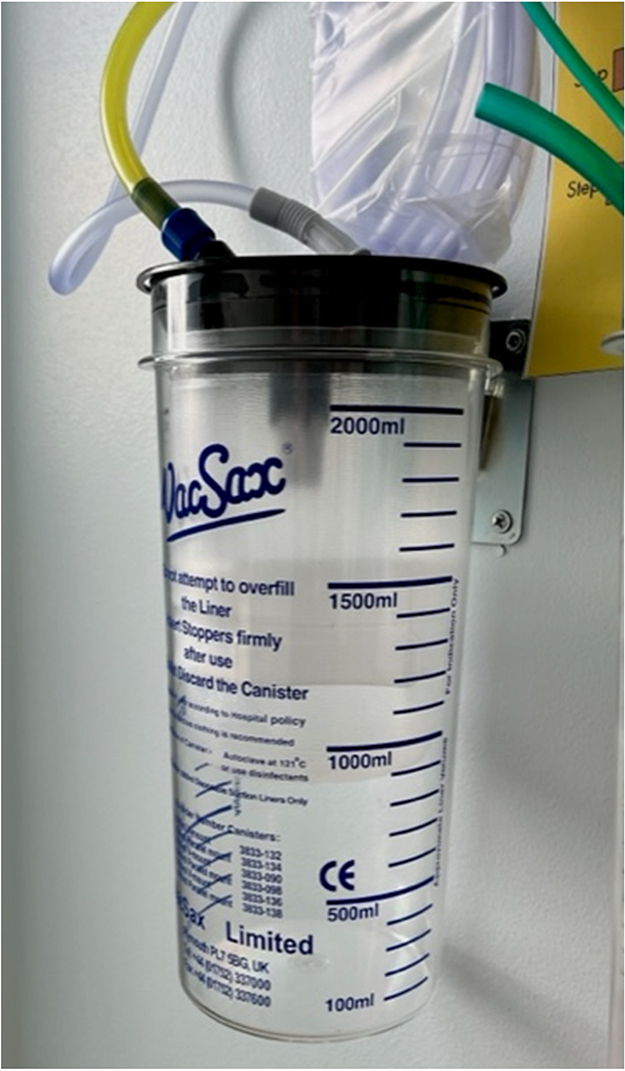
Attachment to wall suction, with visible markings to allow measurement of aspirated fluid

The skin is punctured with the canula and then attached to a three-way tap opened to the 5ml syringe. Using gentle manual vacuum suction via the 5ml syringe, the seroma cavity is entered, as confirmed by a flashback of seroma fluid, and appropriate samples for laboratory examination are prepared. The three-way tap is then turned to the 10ml syringe with one end of the simple suction tubing placed inside the syringe (Figure 2), while the other end is connected to wall suction (Figure 3). The needle is left in the cannula to allow telescoping into deeper aspects of the cavity using a softer tip. Suction strength can be altered depending on the success of aspiration, the viscosity of the aspirate and patient comfort.

## Discussion

Compared with the previously described vacuum wound drainage system, wall suction improves efficiency and reduces the physical strain on the operator's hands. We use a cannula instead of an angiocatheter to allow telescoping, with a softer end reaching the deeper aspects of any cavity. This method is easy to replicate and is more efficient than manual aspirations.
